# Genome-wide12 DNA methylation profiling in the superior temporal gyrus reveals epigenetic signatures associated with Alzheimer’s disease

**DOI:** 10.1186/s13073-015-0258-8

**Published:** 2016-01-19

**Authors:** Corey T. Watson, Panos Roussos, Paras Garg, Daniel J. Ho, Nidha Azam, Pavel L. Katsel, Vahram Haroutunian, Andrew J. Sharp

**Affiliations:** Department of Genetics and Genomic Sciences, Icahn School of Medicine at Mount Sinai, New York, NY USA; Department of Psychiatry, Icahn School of Medicine at Mount Sinai, New York, NY USA; Mental Illness Research, Education, and Clinical Center (VISN 3), James J. Peters VA Medical Center, Bronx, NY USA; Department of Neuroscience, Icahn School of Medicine at Mount Sinai, New York, NY USA

## Abstract

**Background:**

Alzheimer’s disease affects ~13 % of people in the United States 65 years and older, making it the most common neurodegenerative disorder. Recent work has identified roles for environmental, genetic, and epigenetic factors in Alzheimer’s disease risk.

**Methods:**

We performed a genome-wide screen of DNA methylation using the Illumina Infinium HumanMethylation450 platform on bulk tissue samples from the superior temporal gyrus of patients with Alzheimer’s disease and non-demented controls. We paired a sliding window approach with multivariate linear regression to characterize Alzheimer’s disease-associated differentially methylated regions (DMRs).

**Results:**

We identified 479 DMRs exhibiting a strong bias for hypermethylated changes, a subset of which were independently associated with aging. DMR intervals overlapped 475 RefSeq genes enriched for gene ontology categories with relevant roles in neuron function and development, as well as cellular metabolism, and included genes reported in Alzheimer’s disease genome-wide and epigenome-wide association studies. DMRs were enriched for brain-specific histone signatures and for binding motifs of transcription factors with roles in the brain and Alzheimer’s disease pathology. Notably, hypermethylated DMRs preferentially overlapped poised promoter regions, marked by H3K27me3 and H3K4me3, previously shown to co-localize with aging-associated hypermethylation. Finally, the integration of DMR-associated single nucleotide polymorphisms with Alzheimer’s disease genome-wide association study risk loci and brain expression quantitative trait loci highlights multiple potential DMRs of interest for further functional analysis.

**Conclusion:**

We have characterized changes in DNA methylation in the superior temporal gyrus of patients with Alzheimer’s disease, highlighting novel loci that facilitate better characterization of pathways and mechanisms underlying Alzheimer’s disease pathogenesis, and improve our understanding of epigenetic signatures that may contribute to the development of disease.

**Electronic supplementary material:**

The online version of this article (doi:10.1186/s13073-015-0258-8) contains supplementary material, which is available to authorized users.

## Background

Alzheimer’s disease (AD) is the most common neurodegenerative disorder and the leading cause of dementia in the elderly [1]. Diagnosis of AD is based on the presence of neurofibrillary tangles and amyloid plaques [2], and symptoms typically include memory loss and impaired cognitive ability. Although the pathological hallmarks associated with dementia-related symptoms in AD appear largely similar between both the early-onset and late-onset forms of the disease, their underlying etiologies contrast [3]. Whereas early-onset AD is a familial autosomal dominant disorder caused by rare, highly penetrant mutations in one of a small set of genes (*APP*, *PSEN1*, and *PSEN2*), the more common late-onset form of the disease (accounting for 90–95 % of cases) occurs sporadically, and risk is determined by complex underlying mechanisms [3–6]. Estimates based on twin concordance rates suggest heritability of late-onset AD is as high as 70 %, implicating major roles for genetic as well as non-genetic factors [6]. Indeed, through candidate gene studies, as well as more recent genome-wide association studies (GWASs) and whole-exome sequencing, both common and rare variants associated with the late-onset form of AD have been identified [7–11]. Collectively, however, common GWAS variants account for only a modest proportion (~30 %) of the underlying variance in disease susceptibility [12]. Several environmental factors are also thought to play a role [5, 6], yet exactly how these contribute to risk, onset, and progression remains poorly defined.

Recently, there has been increasing interest in the role of epigenetic mechanisms in the interaction between the genome and environment in human diseases [13, 14], including AD [15, 16]. Epigenetic alterations can be defined as modifications to DNA that impact gene expression and phenotype without a change in the nucleotide sequence. These changes can arise within cells of an individual and be maintained through mitosis [17], as well as passed from parent to offspring meiotically [18]. One of the best-studied epigenetic modifications involves changes in DNA methylation at CpG dinucleotides. The establishment of DNA methylation is essential for normal cell development and differentiation [19], and impacts many key cellular processes, including gene regulation [20], X chromosome inactivation [21, 22], and genomic imprinting [23]. The application of genome-wide methylation profiling techniques has led to a rapid increase in the characterization of methylation patterns across the genome, and has facilitated the identification of differentially methylated regions (DMRs) associated with evolutionary processes [24], human aging [25–29], cancer [30, 31], and complex disease [32–35].

Several lines of evidence point to the influence of DNA methylation in AD pathogenesis [15], including direct connections between AD and DNA methylation that have been observed both globally and at specific loci. For example, differences in tissue-wide methylation patterns in disease-relevant brain regions have been reported in patients with AD compared to controls, as well as in monozygotic twins discordant for AD [36, 37]. Locus-specific examples from targeted gene studies also demonstrate a role for DNA methylation changes in AD and include observed disease-associated differences at ribosomal RNA gene promoters [38], genome-wide LINE-1 elements [39], and known AD susceptibility genes [40]. More recently, three epigenome-wide studies (EWAS) have been conducted in AD, collectively generating DNA methylation profiles from three different brain regions of patients with AD [41–43], observing both cross-tissue and tissue-specific effects. Importantly, each of these studies discovered differentially methylated CpGs outside of well-established AD genetic risk loci, highlighting the potential utility of EWAS in the characterization of novel genes and pathways underlying disease processes.

In the present study, we have used the Illumina Infinium HumanMethylation450 array platform to conduct a genome-wide screen of DNA methylation in the superior temporal gyrus (STG) of 34 patients with AD and 34 controls, a brain region recently demonstrated to be a site of significant AD-associated gene dysregulation [44]. Building on previous EWAS in AD, which have primarily focused on single CpG analysis, we applied our recently developed pipeline that aims to identify DMRs harboring multiple statistically significant CpGs exhibiting concordant disease-associated changes in methylation [32]. Using this approach we have identified novel and robust DMRs associated with >400 coding transcripts, many of which have known roles in brain function and AD pathology. Additionally, we show that identified DMRs co-localize with other functional epigenetic signatures in brain tissues, overlap with risk loci identified in AD GWASs and previous EWASs, and harbor expression quantitative trait loci (eQTLs) associated with changes in brain gene expression.

## Methods

### Study subjects and sample preparation

Tissue samples from the STG of 34 patients with confirmed late-onset AD and 34 non-demented controls matched by age of death (AOD), race, and gender were obtained from the Mount Sinai Brain Bank (www.mssm.edu/research/labs/neuropathology-and-brain-banking). Each donor had previously undergone a battery of pathological evaluations, and diagnosis of AD was based on both clinical and neuropathological criteria [44] (Additional file 1: Table S1). Patients with AD had a mean clinical dementia rating of 3.3, a Braak Stage score average of 5.7 (see [44] for staging classification), a mean cortical plaque density of 19.8 based on measurements from five cortical brain regions [44], and a mean AOD of 79.1 years (range, 66–92 years). Controls were determined to have negligible cortical plaque densities (mean, 0.64), and either no evidence of or only mild clinical symptoms of dementia (mean clinical dementia rating, 0.87; mean Braak Stage score, 1.4), with a mean AOD of 80.5 years (range, 66–95 years). Diagnostic and dementia assessment consent procedures were approved by the institutional review boards of Mount Sinai Medical Center, Jewish Home and Hospital, and the JJ Peters VA Medical Center. Consents for brain donation were obtained in writing from the legal next of kin of all donors.

Tissue dissections and sample preparations were carried out following previously published protocols (see [44–47]). Briefly, following dissections, samples were subjected to proteinase K digestion and treatment with RNAse A. Genomic DNA was then isolated using standard phenol/chloroform extraction and ethanol precipitation methods.

### Infinium HumanMethylation450 BeadChip processing

One microgram of DNA from each sample was sodium bisulfate-treated using the EZ DNA Methylation Kit (Zymo Research, Irvine, CA, USA) and processed for analysis on the Illumina Infinium HumanMethylation450 (Illumina, San Diego, CA, USA) array platform at the Mount Sinai Icahn School of Medicine genomics core facility (New York, NY, USA). Subjects were distributed across six BeadChips (12 samples/array) taking into account AOD, gender, race, and case–control status to mitigate anomalies resulting from potential batch effects. The GenomeStudio Methylation Module Package (version 1.9, Illumina) was used for initial data processing, allowing for the calculation of methylation values (expressed as β-values, ranging from 0 to 1) and detection *P*-values for 482,421 individual probes spanning the 22 autosomes and sex chromosomes. Owing to differences in sex chromosome number between males and females, and the fact that our cohort was of mixed gender, only autosomal loci were considered here.

Before proceeding to statistical analysis, data were processed further following the pipeline developed by Huynh et al. [32]. Probes meeting the following criteria known to impact array performance were excluded: (1) those mapping to more than one position in the human reference genome (build NCBI36; hg18) using BSMAP [48], allowing a maximum of two mismatches and three gaps; and (2) those probes for which a 1000 Genomes Project [49, 50] single nucleotide polymorphism (SNP; minor allele frequency ≥ 0.05) mapped to within 5 base pairs (bp) of the probe-targeted CpG. In addition, on a per sample basis, individual β-values for a given CpG were not considered if their detection *P*-value was > 0.01. Data for the 461,272 remaining autosomal CpGs passing our exclusion criteria in the 68 individuals were color and background adjusted, and quantile normalized using *lumi* and *methylumi*, implemented in R [51, 52] (www.R-project.org). The Beta Mixture Quantile Method, as implemented in BMIQ version 1.3 [53], was also applied to the data to correct Infinium I/II probe type bias. Plots from principal component analysis using autosomal methylation profiles from the 68 samples did not reveal major batch effects or anomalous samples. Raw and processed data for all samples have been deposited in GEO under accession GSE76105.

### Identification of DMRs associated with Alzheimer’s disease in the superior temporal gyrus

We first employed linear regression to delineate disease-specific effects on methylation between cases and controls at each of the 461,272 autosomal CpGs. Linear models were developed to account for various independent variables in addition to disease status, including AOD, gender, race, array/batch, and neuronal/glial cell composition. Post-mortem interval was not included in the test model because there was no significant difference observed between cases and controls (t-test, *P* = 0.881). Cell proportions were estimated from our bulk tissue samples using the CETS R package, developed from 450 K array profiles of sorted neuronal and glial cell subsets from 59 adult individuals [54]. When evaluating results of linear regression, neuronal proportion (NP) was considered only for 154,874 CpGs previously reported to be differentially methylated between the two cell types [54]. For these CpGs, we used the following model: β = AOD + gender + race + array + NP + disease status. For all remaining CpGs, NP was not included in the model. CpGs exhibiting average increases in methylation within the AD group as compared to controls (based on regression coefficients associated with disease status) were defined as hypermethylated (hyper) and those exhibiting decreases as hypomethylated (hypo).

Significant CpGs were clustered into DMRs using a 1 kilobase (kb) sliding window, modified from the genome-tiling method described by Bock [55], and previously developed and implemented by Huynh et al. [32]; a window size of 1 kb was previously shown to be optimal for Illumina Infinium HumanMethylation450 array data based on correlations between methylation levels of closely neighboring CpGs [32]. Fisher’s method was used to combine one-sided regression *P*-values across neighboring CpGs within a given 1 kb window, while taking into account methylation state (i.e.*,* hyper vs*.* hypo). The positions of CpGs on either end of a significant window demarcated the coordinates of each DMR. Combined *P*-values for each window/DMR were corrected using the Benjamini–Hochberg method for false discovery rate (FDR) [56]; DMRs meeting a 1 % FDR cutoff were used for downstream analyses.

### CpG/DMR annotation and overlap with genomic features

Probes/CpGs were annotated based on their overlap with specific genomic features using BedTools version 2.1 [57]. Features included functional RefSeq (hg18) genes and promoters (defined as ± 2 kb from the transcriptional start site, TSS), where CpGs were considered to be intergenic if they overlapped neither gene bodies nor promoters; CpG islands (CpGi; UCSC/hg18 annotation), shores (±2 kb from CpGi), shelves (±2 kb from CpG shores), and sea (not within islands, shores, or shelves); and DNaseI hypersensitivity sites and histone marks (H3K9ac, H3K27ac, H3K27me3, H3K4me1, and H3K4me3) in various human brain datasets generated as part of the ENCODE and REMC projects and curated as described previously [58, 59].

DMR enrichments in specific gene-related and CpGi-related features were tested using the χ-square test, by comparing the proportions of DMR-CpGs within the genomic features to the overlap of these features with a background list of 461,272 autosomal CpGs from the 450 K array. For instances in which DMRs overlapped both a promoter and gene body, the promoter annotation was given precedent. The enrichment of DMRs overlapping ENCODE and REMC datasets was tested using INRICH [60]; DMRs (hyper and hypo were considered separately) were used as test regions, the curated histone marks were used as target regions, and again the background set of 450 K CpGs was used as the map file. INRICH estimates if the DMRs overlap curated histone marks more than expected by chance; DMRs were permuted within the genome but matched to the associated DMRs in terms of the number of DMR sites and the number of overlapping histone marks. Empirical *P*-values were estimated based on 10,000 permutations.

RefSeq gene promoters (±2 kb TSSs) overlapped by DMRs were assessed for potential enrichments of defined transcription factor binding sites (TFBS) characterized in human lymphoblastoid cell lines (LCLs; *n* = 282 TFBS motifs), and a human medulloblastoma cell line (*n* = 258 TFBS motifs). Specifically, we used LCL TFBS reported by Pique-Regi et al. [61] characterized using the CENTIPEDE algorithm, after removing binding motifs lacking specifically assigned TFs; medulloblastoma TFBSs were defined as sites where evolutionarily conserved binding motifs of human/mouse/rat TFs overlapped regions of open chromatin based on DNaseI/formaldehyde-assisted isolation of regulatory elements/chromatin immunoprecipitation synthesis [58, 62]. The UCSC tracks used to compile the medulloblastoma dataset were found at http://genome.ucsc.edu/cgi-bin/hgFileUi?db=hg19&g=wgEncodeOpenChromSynth and http://hgdownload.cse.ucsc.edu/goldenPath/hg19/database/tfbsConsSites. Enrichments were calculated by comparing the counts of TFBSs within our set of DMR-associated promoters to the number of counts occurring in a background list of all RefSeq promoters overlapped by all sampled 450 K probes. The significance of enrichments was assessed using Fisher’s exact test (*P*-values were Bonferonni corrected, with a threshold set to *P* < 0.01), including only motifs found in at least 5 % of the 276 tested DMR-associated gene promoters. Gene ontology (GO) enrichments for DMR-associated genes were assessed using GOrilla [63].

### Assessing DMRs in the context of GWAS SNPs and brain eQTLs

The GWAS SNPs used were downloaded from the NHGRI GWAS Catalog [64], using entries under “Alzheimer’s Disease” (access date: December 2014), including SNPs recently reported in a large meta-analysis [11]; only SNPs with *P*-values < 10^−6^ were considered. To generate brain eQTLs, we used the gene expression and genotyping datasets Braincloud [65] [GEO accession number: GSE30272], NIA/NIH [66] [GEO accession number: GSE15745], Harvard Brain Tissue Resource Center [67] [GEO accession number: GSE44772], and UK Brain Expression Consortium [68] [GEO accession number: GSE46706]. Brain eQTLs were determined using methods published previously [69]. To detect the overlap among the index GWAS AD SNPs and eQTLs we used the regulatory trait concordance (RTC) approach [70, 71]. The RTC method detects the overlap of disease-associated variants with functional SNPs, accounting for the correlation structure in the genome (i.e., linkage disequilibrium, LD). RTC scores range from 0 to 1, with values ≥0.9 indicating likely causal regulatory effects, as demonstrated previously [70, 71]. For downstream analysis described here we consider pairs of AD GWAS SNP–eQTLs with RTC ≥0.9. The enrichment analysis of DMRs with AD-associated eQTLs was conducted using the GoShifter package (https://www.broadinstitute.org/mpg/goshifter/). GoShifter estimates the significance of overlap between trait-associated variants (AD-associated eQTLs) and epigenome annotations (DMRs), by generating null distributions of randomly shifting annotations locally within a tested region. For this analysis we used 10,000 permutations.

### Technical validation of CpG methylation

Primers for locus-specific Sequenom MassARRAY EpiTYPER (Sequenom, San Diego, CA, USA) assays were designed using the EpiDesigner primer design software (http://www.epidesigner.com/start3.html). The same bisulfite-converted DNA samples used for array analysis were used for EpiTYPER PCR amplification following manufacturer’s specifications, and post-PCR sample processing and imaging were carried out at the Einstein College of Medicine Genomics Core (New York, NY, USA). β-values generated by the two technologies were compared using the Pearson’s correlation coefficient (*r*).

## Results and discussion

### Identification of DMRs in the superior temporal gyrus of patients with Alzheimer’s disease

Although many brain regions are affected throughout the progression of AD, an extensive study of gene expression changes associated with late-onset AD severity across 15 brain regions recently found the STG to be a site of significant gene dysregulation [44], motivating our focus on this specific region in the present study. We conducted genome-wide profiling of DNA methylation in STG bulk tissue samples from 34 patients with AD and 34 non-demented controls. Quality control processing (see “Methods”) ultimately resulted in high-quality methylation data for 461,272 autosomal CpGs in each of the 68 individuals for differential methylation analysis. Before proceeding to tests for differential methylation, we tested the reproducibility of our 450 K array data by assessing the extent of technical variation at CpGs within five genomic regions using independent locus-specific EpiTYPER PCR-based assays in 30–55 individuals from our cohort. Methylation estimates for the six CpGs tested on both platforms were significantly correlated between the two technologies (*P <* 0.005; Additional file 2: Table S2).

Recent studies of genome-wide DNA methylation have revealed considerable effects of age [25–29], gender [72], ethnicity [73], and cellular composition [19, 54, 74, 75]. Although our disease and control samples were relatively well matched for AOD, gender, and ethnicity, our estimations of neuronal versus glial cell proportions revealed a smaller proportion of neuronal cells in our AD samples (AD mean = 0.247; Control mean = 0.303; t-test, *P =* 0.00099; Additional file 3: Figure S1). This is consistent with histological studies reporting neuronal loss in the brains of patients with AD [76]. Thus, we used multivariate linear regression to delineate significant AD-associated effects on DNA methylation while accounting for potential effects of these variables. It is worth noting that when we repeated our analysis without considering neuronal proportions using a t-test (data not shown), we found that a large fraction of the CpGs that were significantly associated with AD (~62 %) were also found to have significant differences in methylation (>5 %) between neuronal and glial cells [54], demonstrating the importance of incorporating cell composition information into methylation studies in DNA extracted from bulk tissue.

We first compared results from our linear regression analysis to the top 100 CpGs recently reported from an EWAS conducted in STG tissue in a separate cohort of patients with AD and controls [42]. Of the 96 CpGs also screened in our study, 22 (22.9 %) were found to be differentially methylated with the same directional change in patients with AD in our cohort (*P* < 0.05, one-tailed). We also assessed the degree of methylation differences associated with disease in our samples compared to the Lunnon et al. [42] cohort for these same 96 CpGs, and observed a significant correlation between the two datasets (*r* = 0.34; *P* = 0.00067; Additional file 4: Figure S2). The extent of replication observed between our two cohorts is comparable to that initially reported by Lunnon et al. [42] between their samples and other independent cohorts. The incomplete overlap across studies is to be expected given the smaller sample sizes studied to date. Analogous to what has been observed in genetic studies such as GWAS, with increases in cohort sizes, we should expect to see stronger and broader reproducibility of EWAS results.

**Fig. 1:**
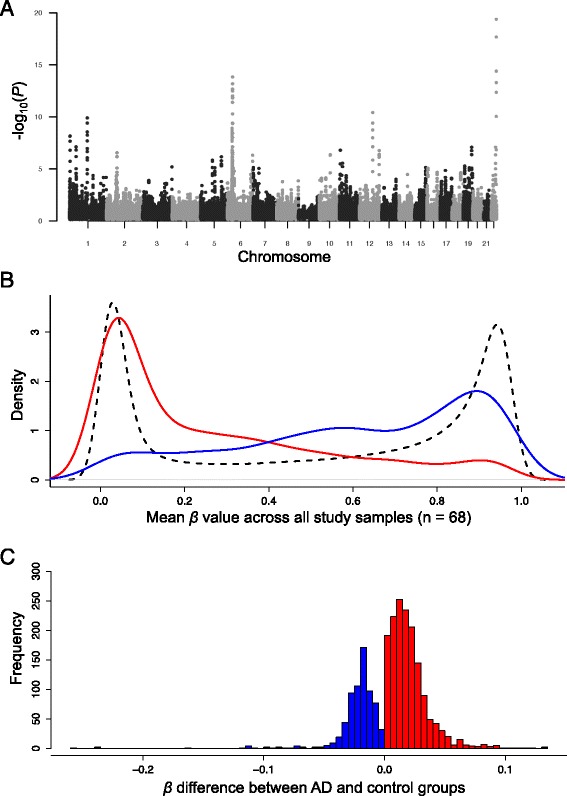
Genome-wide distribution and characteristics of differentially methylated regions identified in the superior temporal gyrus of patients with Alzheimer’s disease (*AD*). **a** Manhattan plot showing -log_10_ *P*-values from the sliding-window analysis of autosomal CpGs in AD cases versus controls. **b** Comparison of population level mean β-values for hypomethylated (*blue*, *n* = 1,260) and hypermethylated (*red*, *n* = 3,395) CpGs, and background CpGs (*black*; all autosomal CpGs screened, *n* = 461,272). **c** Distribution of per CpG mean β-value changes associated with AD status (>0, *red*; <0, *blue*) estimated by multiple regression, after regressing out effects of age, gender, race, and neuronal/glial cell proportions. Only data for CpGs within DMRs that were independently significant by linear regression (*P <* 0.05; *n* = 2,220) are plotted

To increase power in EWAS using smaller disease cohorts, several methods have recently been developed to extend beyond single CpG analysis by leveraging concordant statistical signals from neighboring CpGs to identify DMRs [55, 77]. For our primary analysis, we paired linear regression with a 1 kb sliding window method [32] to search for regions of the genome containing clusters of CpGs exhibiting similar changes in methylation with disease, limiting the likelihood of identifying false positives and allowing for the identification of more robust DMRs. The distribution of all tested autosomal CpGs and DMR-associated Fisher’s *P*-values (FDR-corrected) are displayed in Fig. 1a. Based on a 1 % FDR cutoff, we identified 479 DMRs, with an average size of 927 bp (Additional file 5: Figure S3A). In total, these DMRs included 4,565 CpGs, 48 % of which were independently significant based on linear regression (*P* < 0.05, one-tailed), with an average of 4.63 significant CpGs per DMR (min = 1, max = 24; Additional file 5: Figure S3B). Summary data and annotation for all DMR-CpGs are provided in Additional file 6: Table S3.

Globally, population-wide (AD and controls) β-value averages across all 461,272 autosomal CpGs showed a bimodal distribution (Fig. 1b), with the majority of values falling either below 0.2 or above 0.8. The DMRs identified in our study were strongly biased toward hypermethylated changes (increased in AD; hyper-DMRs = 321, hypo-DMRs = 158; Fig. 1c). Given that AD is linked to aging, it is interesting that CpG DNA methylation has also been shown to increase with age in multiple studies of the human brain [25, 28]. Significant AD-associated CpG methylation was also recently reported to independently correlate with age [41]. We further investigated potential links between AD-DMR CpGs and aging in our dataset by assessing the effects of sample AOD on methylation at the top significant CpG within each DMR (hyper, *n* = 321; hypo, *n* = 158) in control samples (*n* = 34; ages = 66–95), again using multivariate linear regression to account for effects of gender, ethnicity, array/batch, and neuronal/glial proportions. Of the hypermethylated DMR-CpGs, ~21.8 % were significantly associated with AOD (*P* < 0.05), compared to only ~12 % of hypomethylated DMR-CpGs (Additional file 7: Figure S4A). The degree of significance (−log_10_*P*-value) and absolute estimated regression coefficients were also higher on average for hypermethylated DMR-CpGs (Additional file 7: Figure S4B).

Similar to recent reports in AD and other complex diseases [32–35, 41–43], excluding cancer, the average effect of disease state on CpG methylation was modest (Fig. 1c), with an average absolute β-value change of 0.021 at significant CpGs within DMRs. When only the top CpG per DMR with respect to β-value change was considered, this mean difference increased slightly to 0.03. Importantly, however, even modest differences in methylation have been shown to associate with significant alterations in gene expression [32, 34, 41–43].

**Fig. 2:**
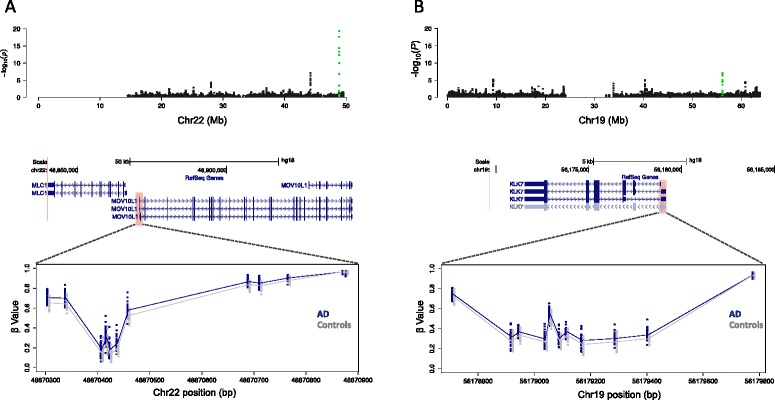
Neighboring CpGs within significant differentially methylated regions (DMRs) exhibit consistent between-group differences in DNA methylation. Zoomed -log_10_ *P*-value plots for chromosome 22 (**a**, *upper panel*) and chromosome 19 (**b**, *upper panel*). The positions of significant DMRs corresponding to plotted green points in (**a**) and (**b**) are shown in the context of RefSeq gene annotations (hg18) for promoter regions of *MOV10L1* (**a**, *middle panel*) and *KLK7* (***b***, *middle panel*). The approximate positions of each DMR are indicated by *red shaded boxes*. Detailed images for each locus (*lower panels*) showing individual CpG β-values, with Alzheimer’s disease (*AD*) samples shown in *blue* and control samples in *gray*. The AD and control group mean β-values are indicated by solid *blue* and *gray lines*, respectively. *Mb* megabase

Nonetheless, despite modest methylation differences, we observed consistent changes amongst neighboring CpGs within DMRs. For example, genomic regions for two DMRs are plotted in Fig. 2, illustrating consistent AD versus control group differences across each locus. The 25 most significant DMRs by FDR-corrected *P*-value and associated data are shown in Table 1, including the physical relationship of each DMR to RefSeq gene annotations. Eight of the genes overlapped by these top 25 DMRs (*LOC100507547*, *PPT2*, *PPT2-EGFL8*, *PRDM16*, *PRRT1*, *C10orf105*, *CDH23*, and *RNF39*) were also among genes recently reported to be associated with the most significantly differentially methylated CpGs in one or more of three brain regions (entorhinal cortex, prefrontal cortex, or STG) in patients with AD [42, 43].

**Table 1:** Top 25 differentially methylated regions associated with Alzheimer’s disease in the superior temporal gyrus

Chr	Start, hg18	End, hg18	DMR length	Number of CpGs	Associated genes	DMR *P*-value	DMR state	Largest β difference	Most significant *P* value^a^	CpG ID^b^
chr22	48870305	48871199	894	13	*MOV10L1*	4.26E-20	Hyper	0.069	1.19E-06	cg10828284
chr6	33352149	33354467	2318	53	*B3GALT4*	1.48E-14	Hyper	0.03	0.00141	cg13882090
chr12	88267918	88269805	1887	13	*DUSP6*	3.94E-11	Hyper	0.045	7.58E-05	cg05769889
chr1	119332644	119334449	1805	18	*TBX15*	1.27E-10	Hypo	0.039	0.00207	cg03942051
chr6	30202278	30203782	1504	31	*intergenic (nearest gene, TRIM40)*	1.28E-09	Hyper	0.03	0.00084	cg08548396
chr6	30081535	30083688	2153	53	*HLA-J, ZNRD1-AS1*	3.65E-09	Hyper	0.05	0.00192	cg05187508
chr1	3181078	3183141	2063	12	*PRDM16*	6.99E-09	Hypo	0.038	6.17E-05	cg19263228
chr1	43606133	43607345	1212	12	*ELOVL1*	7.72E-08	Hypo	0.023	0.00037	cg06350161
chr22	44187332	44188708	1376	17	*RIBC2, SMC1B*	8.38E-08	Hyper	0.037	0.0008	cg22884516
chr19	56178712	56179781	1069	11	*KLK7*	8.38E-08	Hyper	0.038	0.0003	cg27497839
chr11	5573477	5574985	1508	10	*TRIM6, TRIM6-TRIM34*	1.64E-07	Hyper	0.098	0.001	cg00375457
chr12	131575796	131576836	1040	30	*FBRSL1*	1.79E-07	Hyper	0.028	0.00117	cg25694349
chr6	32252644	32254758	2114	39	*AGPAT1, RNF5, RNF5P1*	1.79E-07	Hypo	0.032	0.00267	cg11043450
chr2	70977195	70981085	3890	32	*VAX2*	2.83E-07	Hypo	0.021	0.0005	cg03711129
chr6	32225354	32230126^c^	4611	70	*LOC100507547, PPT2, PPT2-EGFL8, PRRT1*	3.42E-07	Hyper	0.024	0.00044	cg01111041
chr10	73148990	73149855	865	6	*C10orf105, CDH23*	4.38E-07	Hypo	0.034	0.00036	cg18668540
chr6	168159909	168161482	1573	15	*KIF25*	4.91E-07	Hyper	0.063	0.00055	cg03873264
chr5	139207501	139208427	926	8	*NRG2*	7.06E-07	Hyper	0.035	1.63E-05	cg15992535
chr6	30146232	30147781	1549	40	*RNF39*	1.33E-06	Hyper	0.046	0.034	cg03293507
chr5	79021180	79021917	737	12	*CMYA5*	1.48E-06	Hyper	0.03	0.00351	cg23279355
chr13	32120874	32125755	4881	70	*TNXB*	2.43E-06	Hypo	0.025	0.00034	cg15786918
chr11	20089216	20089500	284	2	*NAV2*	2.93E-06	Hypo	0.032	1.41E-09	cg12711760
chr6	32912605	32914526	1921	28	*TAP2*	4.02E-06	Hyper	0.032	0.00039	cg03438552
chr19	9334057	9335129	1072	13	*ZNF177, ZNF559-ZNF177*	5.40E-06	Hyper	0.019	2.04E-05	cg17283453
chr6	30819377	30821226	1849	44	*FLOT1,IER3*	5.65E-06	Hyper	0.031	0.00012	cg13137376

^a^ *P*-value from per CpG linear regression before DMR/sliding-window analysis

^b^CpG corresponding to most significant *P*-value in DMR (^a^)

^c^DMR encompassing single nucleotide polymorphisms in linkage disequilibrium with Alzheimer’s disease genome-wide association study loci/brain expression quantitative trait loci (see also Additional file 11: Table S7)

*DMR* differentially methylated region

**Fig. 3:**
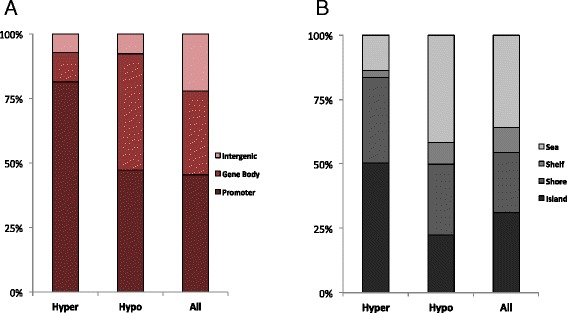
Summary characteristics of differentially methylated region (DMR) CpGs in the context of gene and CpG island features. Proportion of CpGs within hypermethylated DMRs, hypomethylated DMRs, and background CpG sets that fall within various gene (**a**) and CpG island (**b**) feature annotations

In total, we found that ~92 % of DMR-CpGs directly overlapped RefSeq gene transcript coordinates. However, their distribution within different gene features depended on methylation state. For example, compared to the distribution of all 450 K array CpGs, CpGs within hyper-DMRs were more commonly found in gene promoters (±2 kb TSSs), whereas CpGs in hypo-DMRs were enriched in the gene body of RefSeq transcripts (Fig. 3a). CpGs in hyper-DMRs also showed preferential overlap with CpGi’s (Fig. 3b).

### Analysis of DMRs in the context of gene ontology and functional genomic datasets in the human brain

The top DMR in our dataset (Table 1) overlapped the RNA helicase gene *MOV10L1*. Although little is known about the specific function of this gene in the brain, many other genes associated with top DMRs have reported roles in brain function, such as *RNF39*, *KLK7*, *DUSP6*, *NAV2*, and *NRG2*. In the context of AD pathology, the protein KLK7 (Fig. 2b; Table 1) was recently shown to cleave and degrade β-amyloid (Aβ) and mitigate Aβ-mediated toxicity in vitro [78], possibly consistent with the observed negative correlation between *KLK7* expression and AD disease severity [79]. *DUSP6* was recently shown to be a target of the AD-associated microRNA miR-125b, exhibiting decreased expression in AD brains; notably, knockdown of *DUSP6* in primary hippocampal neurons lead to a significant increase in tau protein hyperphosphorylation, a key hallmark of AD [80].

It is also interesting that several of the top DMR-associated genes are involved in adiposity, fat distribution, and the synthesis and metabolism of cholesterol and lipids (*PRDM16*, *TBX15*, *ELOVL1*, and *AGPAT1*) [81, 82]. GO enrichments in categories related to cholesterol/lipid metabolism are among the highest observed for AD risk genes identified by GWAS [83]; alterations in the expression of related genes have been observed previously in the same cohort studied here [84–86]. Experimental data also highlight the potential importance of genes involved in these processes in AD pathology, with alterations in lipid and cholesterol levels having been observed in the blood, cerebrospinal fluid, and brains of patients with AD [87, 88], and associated with cognitive performance [89]. Furthermore, at the molecular level, both cholesterol and lipids have important roles in modulating the production and aggregation of Aβ via interactions with well-known mediators of AD pathogenesis and risk, such as APP, APOE, PSEN1, and BACE1 [90, 91].

**Fig. 4:**
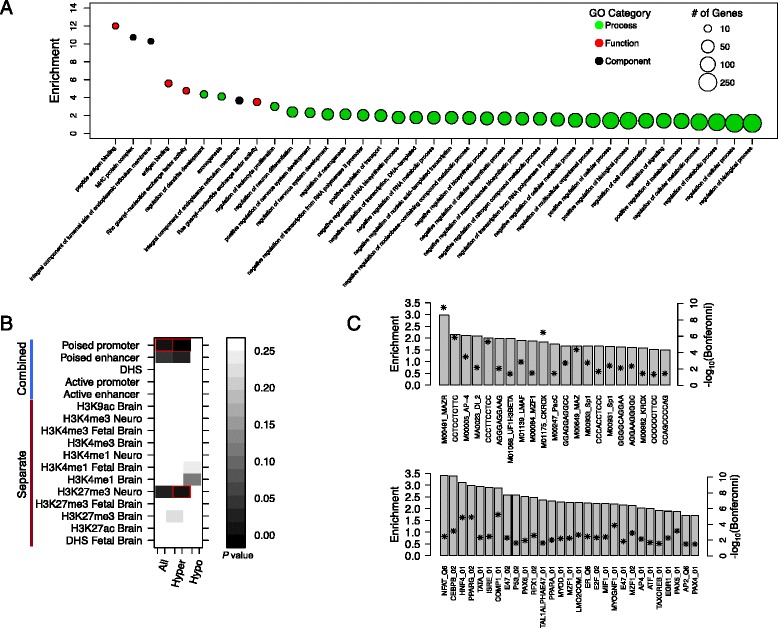
Differentially methylated genes (DMRs) are enriched in genes and functional genomic regions with relevance to Alzheimer’s disease pathology. **a** Summary of gene ontology (*GO*) term enrichments for DMR-associated genes (*n* = 475). Significantly enriched terms (*q <* 0.2) are ranked by estimated fold-enrichment, and colored according to their broader classification (“Molecular Function,” “Biological Process,” and “Cellular Component”). The number of DMR-associated genes (min = 6; max = 268) categorized in each GO term are indicated by circle size. **b** Heat map of raw *P*-values associated with enrichment analysis DMRs in regions characterized by individual and combined histone signatures in adult and fetal brain/neuron datasets. *Red boxes* indicate significant enrichments after correction (corrected *P* < 0.05). **c** Significant enrichments of transcription factor binding site motifs within DMR-associated RefSeq gene promoters, based on analysis of datasets curated separately in a medulloblastoma cell line (*upper panel*) and a lymphoblastoid cell line (*lower panel*). Motifs are labeled along the *x-axis*, and are shown in ranked order in each panel according to fold-enrichment. Bonferroni-corrected -log_10_ *P*-values are shown as *asterisks*

To more broadly explore the potential function of genes overlapped by AD-associated DMRs, we conducted GO analysis using a list of 475 RefSeq genes containing DMRs within their promoters and/or gene bodies. After FDR correction (*q <* 0.2) and the removal of terms associated with five or fewer genes, compared to a background list of RefSeq genes overlapped by CpGs found on the 450 K array, DMR-associated genes were enriched for 30 GO terms linked to biological processes, three GO terms linked to cellular components, and four GO terms linked to molecular function (Fig. 4a; Additional file 8: Table S4). Significant GO terms included “regulation of neuron differentiation” (*P =* 2.93 × 10^−5^, enrichment = 2.39), “axonogenesis” (*P =* 1.58 × 10^−4^, enrichment = 4.12), and “regulation of neurogenesis” (*P =* 8.53 × 10^−5^, enrichment = 2.13), associated with biological processes that point to roles of DMR-genes in the development of neurons and other cells in the nervous system. In addition, as noted for several genes in the top DMR list, multiple ontology terms associated with cellular metabolism were also enriched for DMR-associated genes (Fig. 4a; Additional file 8: Table S4).

We next assessed the association of our DMRs with functional genome-wide datasets generated in non-diseased brain tissue/cell lines. During development and cellular differentiation, DNA methylation is known to act in concert with chromatin alterations such as histone methylation and acetylation to modify gene expression programs [92]. Furthermore, the occurrence of histone modifications can illuminate genomic regions with functional properties in the context of disease [69]. Thus, to explore whether our DMRs overlapped functional regions relevant to the human brain, we tested for enrichment of AD DMRs in regions with repressive and permissive histone modification profiles (H3K9ac, H3K27ac, H3K27me3, H3K4me1, and H3K4me3) generated from fetal and adult bulk brain tissue, as well as iPS-derived neurons (Fig. 4b; Additional file 9: Table S5). We found significant enrichments for hyper DMRs in poised promoters (corrected *P* = 0.001), also referred to as bivalent domains, characterized by the occupancy of H3K27me3 and H3K4me3. Bivalent domains are generally thought to take on repressed states, while remaining “poised” for activation, and these regions are known to have important roles in cell development and pluripotency [93]. It is interesting to note that, although CpGs within poised promoters are typically characterized by hypomethylation, increased DNA hypermethylation associated with human aging has been shown to occur preferentially in bivalent domains in various tissues, including the brain [26, 29, 94]; such changes have also been noted in cancer and cell culture, and may suggest that hypermethylation of bivalent domains results in a reduction of cell pluripotency [95, 96]. Given the strong connection between AD and aging, the overlap observed here between DMRs and poised promoters could have important implications for understanding molecular mechanisms underlying disease onset and progression.

DNA methylation is also known to play complex roles in TF binding, in some cases either hindering or facilitating interactions between DNA motifs and proteins [97]. Thus, we tested for enrichment in AD-DMR-associated RefSeq gene promoters (*n* = 276) of TFBS from two independent datasets, one curated from LCLs, and a second brain dataset consisting of evolutionarily conserved TFBSs residing within regions of open chromatin (active) in a medulloblastoma cell line (see “Methods”). After applying a multiple-testing correction and stringent filter (Bonferroni *P* < 0.01), we noted 29 and 28 significant TF motif enrichments for the medulloblastoma and LCL datasets, respectively (Fig. 4c). The strongest enrichments were found for motifs of NFAT in the medulloblastoma dataset (fold-enrichment = 3.45, Bonferroni = 0.003), and MAZR in the LCL dataset (fold-enrichment = 2.97; Bonferroni = 2.18 × 10^−10^); the NFAT transcription factor family, in particular, has demonstrated roles in AD pathology [98, 99]. Several other motifs/TFs known to regulate pathways involved in brain function were also identified, such as PPARG, PPARA, and SP1. Specifically in the context of previous findings in AD, SP1 has been shown to regulate enriched gene sets that exhibit expression changes associated with memory impairment in patients with AD [100].

### Analysis of DMRs in the context of Alzheimer’s disease GWAS SNPs and brain eQTLs

Overlap between loci of differential methylation/expression, methylation QTLs and eQTLs, and GWAS regions has been observed previously in complex disease. Such findings demonstrate that in addition to the ability to identify novel epigenetic signatures underlying risk or disease progression, EWAS data also provide an opportunity to potentially inform the assignment of putative function to genetic variants associated with disease risk, and may help guide functional analyses of GWAS loci [34]. Of the 479 DMRs identified, 15 fell within ±250 kb of a previously reported GWAS SNP, including those associated with *CLU*, *DIP2C*, *FRMD4A*, *HLA*-*DRB1*, *HLA-DQB1*, *CTNNA2*, and *KLK7* (Additional file 10: Table S6); DMRs overlapping promoters of *CLU* and *FRMD4A* fell within ±2 kb of a GWAS SNP.

To further investigate potential functional links with AD GWAS, we integrated DMRs and brain eQTLs with AD GWAS regions, using the regulatory trait approach [70, 71]. We identified 129 risk AD loci that were associated with gene expression of at least one transcript at RTC ≥ 0.9. We examined the enrichment of these AD-associated eQTLs with DMRs using GoShifter. There was no significant enrichment with DMRs (hypomethylated or hypermethylated; empirical *P* > 0.3). However, we found that three AD-associated eQTLs (and SNPs with *r*^*2*^ > 0.8) fell inside of AD-DMRs (Additional file 11: Table S7). This included eQTLs associated with the expression of *AGPAT1*, *TAP2*, and *CLU*. Additional file 12: Figure S5A–C shows spatial relationships between DMRs, GWAS SNPs, and eQTLs. Two of these DMRs encompass SNPs in LD with a single GWAS SNP (rs111418223) but two distinct eQTL signals, both of which impact the expression of genes in the vicinity of the Human Leukocyte Antigen (HLA) gene region. *TAP2* haplotypes have previously been shown to contribute to AD risk via interactions with *APOE4* polymorphisms, with speculated involvement of *TAP2* in connections between herpes simplex virus-1 infection and AD [101]. Both *AGPAT1* and *CLU* have roles in lipid/cholesterol metabolism. Specifically, variants in *AGPAT1* are associated with variation in levels of circulating sphingolipids and phospholipids in human plasma [82], and impairments of both *Agpat1* expression and cholesterol metabolism have also been observed in a rat model of Huntington’s disease [102]. A role for *CLU* in AD pathology has long been suspected, solidified by the identification of variants contributing to AD risk by multiple independent GWAS [103]. Several of these risk variants have been linked to alterations of *CLU* expression and alternative splicing in AD [103–105].

## Conclusions

We have conducted an epigenome-wide screen in the STG of patients with AD to characterize clusters of CpGs exhibiting concordant disease-associated changes in DNA methylation. After accounting for effects of sample age, gender, ethnicity, and neuronal/glial cell proportions, we identified 479 autosomal DMRs, the majority of which were defined by hypermethylation in AD cases compared to controls. Although the degree of average disease-associated methylation differences was relatively modest within DMRs, this observation is consistent with previous findings in AD and other complex disease [41–43], including those shown to influence gene expression at both the transcript and protein levels [32, 41–43].

An important consideration is that, although we accounted for potential effects of cellular composition differences between AD cases and controls in our characterization of differential methylation, additional investigation will be required to assess whether the methylation signatures observed are cell type-specific; this is in fact a limitation of all AD EWAS studies conducted to date using bulk tissue [16]. Despite such limitations, our systems-level analyses of DMRs and associated genes provided evidence for likely roles of these regions in AD pathology. This included observed enrichments of DMR-associated genes for GO terms related to the development and function of neurons, as well as cellular metabolism, both relevant to known molecular and neurological impairments in AD, and consistent with findings from AD transcriptome studies and GWAS [83–86, 106].

We also found that DMRs were distributed non-randomly in the genome, with biases in their co-localization within gene and CpG island annotations, as well as preferential overlap with specific brain histone methylation signatures and gene promoters harboring brain-relevant TFs. Most notably, we found significant enrichments specifically for hypermethylated DMRs in poised promoters of the adult brain; these loci, characterized by the presence of both H3K27me3 and H3K4me3, mark regulatory regions associated with developmental genes that have key roles in cellular differentiation and pluripotency [93, 95]. Poised promoters have also been shown to preferentially overlap CpGs that become hypermethylated with age [26, 94]. Interestingly, we found a strong bias for CpG hypermethylation among AD-DMRs, and that these CpGs were enriched for age-associated methylation changes in our control samples when compared to CpGs within hypomethylated DMRs. Taken together, these points highlight a potential interplay between disease-associated epigenetic alterations and aging in AD pathology, and suggest more targeted research in this area may be warranted.

Finally, our results indicate that the study of epigenetic signatures can aid in the characterization of novel genomic regions associated with disease, particularly those overlooked by alternative approaches. Future challenges in the field include the development of effective strategies for integrating epigenetic and transcriptomic profiles with genetic datasets, as a means to better understand the roles of different forms of variation in AD [16].

## Additional files

Additional file 1: Table S1. Clinical features of AD and control samples. (XLSX 41 kb) Additional file 2: Table S2. Technical replication of six 450 K CpGs using the targeted Sequenom EpiTYPER assays. (XLSX 40 kb) Additional file 3: Figure S1. Estimated neuronal proportions in AD samples are on average lower than those observed in controls. Box plots of per sample neuronal proportions within AD and control sample groups (AD mean = 0.247; control mean = 0.303). Neuronal and glial cell proportions in STG bulk tissue samples were estimated from 450 K methylation profiles of each sample using the CETS R package [54]. A Student’s t-test revealed the difference in neuronal proportions between groups to be significantly different (*P* = 0.00099), motivating our use of per sample neuronal proportions as a covariate in our regression models for identifying regions of differential methylation. (PDF 65 kb) Additional file 4: Figure S2. Comparison of AD-associated methylation changes between two datasets generated from the STG. We compared the estimated mean β-value changes associated with AD status in our dataset (using multiple regression; see “Methods”) to those reported previously for the top 100 differentially methylated CpGs characterized in the STG of an AD discovery cohort by Lunnon et al. [42]. Regression analysis reveals a statistically significant relationship between case–control β-value differences observed in the two studies (*r* = 0.34; *P* = 0.00067), with the majority of compared CpGs showing concordant directional changes in methylation associated with AD case status. The red line represents the line-of-best-fit estimated using linear regression. (PDF 98 kb) Additional file 5: Figure S3. Summary characteristics of significant DMRs identified in the SGT of AD patients. (A) The length in bp of 479 significant DMRs, ranked by size (minimum = 2 bp; mean = 927 bp; maximum = 4,881 bp). (B) The number of CpGs per DMR that were independently significant by linear regression (*P* < 0.05; minimum = 1; mean = 4.63; maximum = 24), plotted in ranked order. (PDF 169 kb) Additional file 6: Table S3. CpGs in significant DMRs with annotation. (XLSX 689 kb) Additional file 7: Figure S4. Significant CpGs within hypermethylated DMRs are enriched for sites associated with aging in controls. (A) The proportion of hypermethylated CpGs that are significantly associated with control sample age is greater among CpGs within AD-associated hypermethylated DMRs. Proportions of significant (*P* < 0.05, one-tailed) and non-significant CpGs (*P* > 0.05, one-tailed), as determined by linear regression, within each group (hyper vs. hypo) are indicated. (B) Distributions of –log_10_ *P*-values and absolute regression coefficients for effects of sample AOD on CpG methylation, determined using linear regression, after partitioning by DMR status. Hypermethylated CpGs are shown in red (*n* = 321), and hypomethylated CpGs are shown in blue (*n* = 158). Means for each metric are indicated by red (hyper) and blue (hypo) dotted lines. (PDF 85 kb) Additional file 8: Table S4. Top GO enrichments for genes associated with DMRs. (XLSX 51 kb) Additional file 9: Table S5. Enrichment results for brain histone mark datasets. (XLSX 10 kb) Additional file 10: Table S6. DMRs occurring within 250 kb of a GWAS SNP associated with AD. (XLSX 46 kb) Additional file 11: Table S7. SNPs within DMRs exhibiting strong LD with AD GWAS hits and brain eQTLs. (XLSX 9 kb) Additional file 12: Figure S5. Genomic regions of three AD-DMRs encompassing SNPs in LD with AD-GWAS risk variants and brain eQTLs. Panels (A-C) show genomic regions surrounding the DMRs presented in Additional file 11: Table S7, including positions of DMRs, AD-GWAS risk SNPs, brain eQTLs, and RefSeq gene annotations. Red boxes indicate genes whose expression is influenced by identified eQTLs; names of GWAS variant and eQTL SNP IDs are provided below relevant annotations. (DOCX 15 kb)

## References

[1] Alzheimer’s Association (2012). Alzheimer’s disease facts and figures. Alzheimers Dement.

[2] Braak H, Braak E (1997). Diagnostic criteria for neuropathologic assessment of Alzheimer’s disease. Neurobiol Aging..

[3] Bird TD (2008). Genetic aspects of Alzheimer disease. Genet Med..

[4] Bertram L, Lill CM, Tanzi RE (2010). The genetics of Alzheimer disease: back to the future. Neuron..

[5] Migliore L, Coppedè F (2009). Genetics, environmental factors and the emerging role of epigenetics in neurodegenerative diseases. Mutat Res..

[6] Gatz M, Reynolds CA, Fratiglioni L, Johansson B, Mortimer JA, Berg S (2006). Role of genes and environments for explaining Alzheimer disease. Arch Gen Psychiatry..

[7] Harold D, Abraham R, Hollingworth P, Sims R, Gerrish A, Hamshere ML (2009). Genome-wide association study identifies variants at CLU and PICALM associated with Alzheimer’s disease. Nat Genet..

[8] Bertram L, Tanzi RE (2009). Genome-wide association studies in Alzheimer’s disease. Hum Mol Genet..

[9] Chapman J, Rees E, Harold D, Ivanov D, Gerrish A, Sims R (2013). A genome-wide study shows a limited contribution of rare copy number variants to Alzheimer’s disease risk. Hum Mol Genet..

[10] Lambert J-C, Grenier-Boley B, Harold D, Zelenika D, Chouraki V, Kamatani Y (2013). Genome-wide haplotype association study identifies the FRMD4A gene as a risk locus for Alzheimer’s disease. Mol Psychiatry..

[11] Lambert JC, Ibrahim-Verbaas CA, Harold D, Naj AC, Sims R, Bellenguez C (2013). Meta-analysis of 74,046 individuals identifies 11 new susceptibility loci for Alzheimer’s disease. Nat Genet..

[12] Lee SH, Harold D, Nyholt DR, Goddard ME, Zondervan KT, Williams J (2013). Estimation and partitioning of polygenic variation captured by common SNPs for Alzheimer’s disease, multiple sclerosis and endometriosis. Hum Mol Genet..

[13] Portela A, Esteller M (2010). Epigenetic modifications and human disease. Nat Biotechnol..

[14] Handel AE, Ebers GC, Ramagopalan SV (2010). Epigenetics: molecular mechanisms and implications for disease. Trends Mol Med..

[15] Mastroeni D, Grover A, Delvaux E, Whiteside C, Coleman PD, Rogers J (2011). Epigenetic mechanisms in Alzheimer’s disease. Neurobiol Aging..

[16] Lunnon K, Mill J (2013). Epigenetic studies in Alzheimer’s disease: current findings, caveats, and considerations for future studies. Am J Med Genet B Neuropsychiatr Genet.

[17] Probst AV, Dunleavy E, Almouzni G (2009). Epigenetic inheritance during the cell cycle. Nat Rev Mol Cell Biol..

[18] Guilmatre A, Sharp AJ (2012). Parent of origin effects. Clin Genet..

[19] Ziller MJ, Gu H, Müller F, Donaghey J, Tsai LT-Y, Kohlbacher O (2013). Charting a dynamic DNA methylation landscape of the human genome. Nature..

[20] Bell JT, Pai AA, Pickrell JK, Gaffney DJ, Pique-Regi R, Degner JF (2011). DNA methylation patterns associate with genetic and gene expression variation in HapMap cell lines. Genome Biol..

[21] Sharp AJ, Stathaki E, Migliavacca E, Brahmachary M, Montgomery SB, Dupre Y (2011). DNA methylation profiles of human active and inactive X chromosomes. Genome Res..

[22] Bala Tannan N, Brahmachary M, Garg P, Borel C, Alnefaie R, Watson CT (2014). DNA methylation profiling in X;autosome translocations supports a role for L1 repeats in the spread of X chromosome inactivation. Hum Mol Genet..

[23] Edwards CA, Ferguson-Smith AC (2007). Mechanisms regulating imprinted genes in clusters. Curr Opin Cell Biol..

[24] Hernando-Herraez I, Prado-Martinez J, Garg P, Fernandez-Callejo M, Heyn H, Hvilsom C (2013). Dynamics of DNA methylation in recent human and great ape evolution. PLoS Genet.

[25] Numata S, Ye T, Hyde TM, Guitart-Navarro X, Tao R, Wininger M (2012). DNA methylation signatures in development and aging of the human prefrontal cortex. Am J Hum Genet..

[26] Rakyan VK, Down TA, Maslau S, Andrew T, Yang T-P, Beyan H (2010). Human aging-associated DNA hypermethylation occurs preferentially at bivalent chromatin domains. Genome Res..

[27] Hannum G, Guinney J, Zhao L, Zhang L, Hughes G, Sadda S (2013). resource genome-wide methylation profiles reveal quantitative views of human aging rates. Mol Cell.

[28] Hernandez DG, Nalls MA, Gibbs JR, Arepalli S, van der Brug M, Chong S (2011). Distinct DNA methylation changes highly correlated with chronological age in the human brain. Hum Mol Genet..

[29] Teschendorff AE, Menon U, Gentry-Maharaj A, Ramus SJ, Weisenberger DJ, Shen H (2010). Age-dependent DNA methylation of genes that are suppressed in stem cells is a hallmark of cancer. Genome Res..

[30] Heyn H, Sayols S, Moutinho C, Vidal E, Sanchez-Mut JV, Stefansson OA (2014). Linkage of DNA methylation quantitative trait loci to human cancer risk. Cell Rep..

[31] Stefansson OA, Moran S, Gomez A, Sayols S, Arribas-Jorba C, Sandoval J (2014). A DNA methylation-based definition of biologically distinct breast cancer subtypes. Mol Oncol..

[32] Huynh JL, Garg P, Thin TH, Yoo S, Dutta R, Trapp BD (2014). Epigenome-wide differences in pathology-free regions of multiple sclerosis-affected brains. Nat Neurosci..

[33] Pidsley R, Viana J, Hannon E, Spiers H, Troakes C, Al-Saraj S (2014). Methylomic profiling of human brain tissue supports a neurodevelopmental origin for schizophrenia. Genome Biol..

[34] Van Eijk KR, de Jong S, Strengman E, Buizer-Voskamp JE, Kahn RS, Boks MP (2014). Identification of schizophrenia-associated loci by combining DNA methylation and gene expression data from whole blood. Eur J Hum Genet..

[35] Liu Y, Aryee MJ, Padyukov L, Fallin MD, Hesselberg E, Runarsson A (2013). Epigenome-wide association data implicate DNA methylation as an intermediary of genetic risk in rheumatoid arthritis. Nat Biotechnol..

[36] Mastroeni D, McKee A, Grover A, Rogers J, Coleman PD (2009). Epigenetic differences in cortical neurons from a pair of monozygotic twins discordant for Alzheimer’s disease. PLoS One..

[37] Chouliaras L, Mastroeni D, Delvaux E, Grover A, Kenis G, Hof PR (2013). Consistent decrease in global DNA methylation and hydroxymethylation in the hippocampus of Alzheimer’s disease patients. Neurobiol Aging..

[38] Pietrzak M, Rempala G, Nelson PT, Zheng J-J, Hetman M (2011). Epigenetic silencing of nucleolar rRNA genes in Alzheimer’s disease. PLoS One..

[39] Bollati V, Galimberti D, Pergoli L, Dalla Valle E, Barretta F, Cortini F (2011). DNA methylation in repetitive elements and Alzheimer disease. Brain Behav Immun..

[40] Yu L, Chibnik LB, Srivastava GP, Pochet N, Yang J, Xu J (2014). Association of brain DNA methylation in SORL1, ABCA7, HLA-DRB5, SLC24A4, and BIN1 with pathological diagnosis of Alzheimer disease. JAMA Neurol..

[41] Bakulski KM, Dolinoy DC, Sartor MA, Paulson HL, Konen JR, Lieberman AP (2012). Genome-wide DNA methylation differences between late-onset Alzheimer’s disease and cognitively normal controls in human frontal cortex. J Alzheimers Dis..

[42] Lunnon K, Smith R, Hannon E, De Jager PL, Srivastava G, Volta M (2014). Methylomic profiling implicates cortical deregulation of ANK1 in Alzheimer’s disease. Nat Neurosci..

[43] De Jager PL, Srivastava G, Lunnon K, Burgess J, Schalkwyk LC, Yu L (2014). Alzheimer’s disease: early alterations in brain DNA methylation at ANK1, BIN1, RHBDF2 and other loci. Nat Neurosci..

[44] Haroutunian V, Katsel P, Schmeidler J (2009). Transcriptional vulnerability of brain regions in Alzheimer’s disease and dementia. Neurobiol Aging..

[45] Haroutunian V, Perl D (1998). Regional distribution of neuritic plaques in the nondemented elderly and subjects with very mild Alzheimer disease. Arch Neurol..

[46] Haroutunian V, Purohit D (1999). Neurofibrillary tangles in nondemented elderly subjects and mild Alzheimer disease. Arch Neurol.

[47] Haroutunian V, Davies P, Vianna C, Buxbaum JD, Purohit DP (2007). Tau protein abnormalities associated with the progression of Alzheimer disease type dementia. Neurobiol Aging..

[48] Xi Y, Li W (2009). BSMAP: whole genome bisulfite sequence MAPping program. BMC Bioinformatics..

[49] Abecasis GR, Altshuler D, Auton A, Brooks LD, Durbin RM, Gibbs RA (2010). A map of human genome variation from population-scale sequencing. Nature..

[50] Abecasis GR, Auton A, Brooks LD, DePristo MA, Durbin RM, Handsaker RE (2012). An integrated map of genetic variation from 1,092 human genomes. Nature..

[51] Du P, Kibbe WA, Lin SM (2008). lumi: a pipeline for processing Illumina microarray. Bioinformatics.

[52] Du P, Zhang X, Huang C-C, Jafari N, Kibbe WA, Hou L (2010). Comparison of Beta-value and M-value methods for quantifying methylation levels by microarray analysis. BMC Bioinformatics..

[53] Teschendorff AE, Marabita F, Lechner M, Bartlett T, Tegner J, Gomez-Cabrero D (2013). A beta-mixture quantile normalization method for correcting probe design bias in Illumina Infinium 450 k DNA methylation data. Bioinformatics..

[54] Guintivano J, Aryee MJ, Kaminsky ZA (2013). A cell epigenotype specific model for the correction of brain cellular heterogeneity bias and its application to age, brain region and major depression. Epigenetics..

[55] Bock C (2012). Analysing and interpreting DNA methylation data. Nat Rev Genet..

[56] Benjamini Y, Hochberg Y (1995). Controlling the false discovery rate: a practical and powerful approach to multiple testing. J R Stat Soc Ser B..

[57] Quinlan AR, Hall IM (2010). BEDTools: a flexible suite of utilities for comparing genomic features. Bioinformatics..

[58] Maurano MT, Humbert R, Rynes E, Thurman RE, Haugen E, Wang H (2012). Systematic localization of common disease-associated variation in regulatory DNA. Science..

[59] Zhu J, Adli M, Zou JY, Verstappen G, Coyne M, Zhang X (2013). Genome-wide chromatin state transitions associated with developmental and environmental cues. Cell..

[60] Lee PH, O’Dushlaine C, Thomas B, Purcell SM (2012). INRICH: interval-based enrichment analysis for genome-wide association studies. Bioinformatics..

[61] Pique-Regi R, Degner JF, Pai AA, Gaffney DJ, Gilad Y, Pritchard JK (2011). Accurate inference of transcription factor binding from DNA sequence and chromatin accessibility data. Genome Res..

[62] Good PJ, Guyer MS, Kamholz S, Liefer L, Wetterstrand K, Kampa D (2004). The ENCODE ( ENCyclopedia Of DNA Elements ) Project. Science.

[63] Eden E, Navon R, Steinfeld I, Lipson D, Yakhini Z (2009). GOrilla: a tool for discovery and visualization of enriched GO terms in ranked gene lists. BMC Bioinformatics..

[64] Welter D, MacArthur J, Morales J, Burdett T, Hall P, Junkins H (2014). The NHGRI GWAS Catalog, a curated resource of SNP-trait associations. Nucleic Acids Res.

[65] Colantuoni C, Lipska BK, Ye T, Hyde TM, Tao R, Leek JT (2011). Temporal dynamics and genetic control of transcription in the human prefrontal cortex. Nature..

[66] Gibbs JR, van der Brug MP, Hernandez DG, Traynor BJ, Nalls MA, Lai S-L (2010). Abundant quantitative trait loci exist for DNA methylation and gene expression in human brain. PLoS Genet..

[67] Zhang B, Gaiteri C, Bodea L-G, Wang Z, McElwee J, Podtelezhnikov AA (2013). Integrated systems approach identifies genetic nodes and networks in late-onset Alzheimer’s disease. Cell..

[68] Ramasamy A, Trabzuni D, Guelfi S, Varghese V, Smith C, Walker R (2014). Genetic variability in the regulation of gene expression in ten regions of the human brain. Nat Neurosci..

[69] Roussos P, Mitchell AC, Voloudakis G, Fullard JF, Pothula VM, Tsang J (2014). A role for noncoding variation in schizophrenia. Cell Rep..

[70] Nica AC, Montgomery SB, Dimas AS, Stranger BE, Beazley C, Barroso I (2010). Candidate causal regulatory effects by integration of expression QTLs with complex trait genetic associations. PLoS Genet..

[71] Grundberg E, Small KS, Hedman ÅK, Nica AC, Buil A, Keildson S (2012). Mapping cis- and trans-regulatory effects across multiple tissues in twins. Nat Genet..

[72] Boks MP, Derks EM, Weisenberger DJ, Strengman E, Janson E, Sommer IE (2009). The relationship of DNA methylation with age, gender and genotype in twins and healthy controls. PLoS One..

[73] Heyn H, Moran S, Hernando-Herraez I, Sayols S, Gomez A, Sandoval J (2013). DNA methylation contributes to natural human variation. Genome Res..

[74] Koestler DC, Christensen B, Karagas MR, Marsit CJ, Langevin SM, Kelsey KT (2013). Blood-based profiles of DNA methylation predict the underlying distribution of cell types: a validation analysis. Epigenetics..

[75] Kozlenkov A, Roussos P, Timashpolsky A, Barbu M, Rudchenko S, Bibikova M (2014). Differences in DNA methylation between human neuronal and glial cells are concentrated in enhancers and non-CpG sites. Nucleic Acids Res..

[76] Gómez-Isla T, Hollister R, West H, Mui S, Growdon JH, Petersen RC (1997). Neuronal loss correlates with but exceeds neurofibrillary tangles in Alzheimer’s disease. Ann Neurol..

[77] Robinson MD, Kahraman A, Law CW, Lindsay H, Nowicka M, Weber LM (2014). Statistical methods for detecting differentially methylated loci and regions. Front Genet..

[78] Shropshire TD, Reifert J, Rajagopalan S, Baker D, Feinstein SC, Daugherty PS (2014). Amyloid β peptide cleavage by kallikrein 7 attenuates fibril growth and rescues neurons from Aβ-mediated toxicity in vitro. Biol Chem..

[79] Bossers K, Wirz KTS, Meerhoff GF, Essing AHW, van Dongen JW, Houba P (2010). Concerted changes in transcripts in the prefrontal cortex precede neuropathology in Alzheimer’s disease. Brain.

[80] Banzhaf-Strathmann J, Benito E, May S, Arzberger T, Tahirovic S, Kretzschmar H (2014). MicroRNA-125b induces tau hyperphosphorylation and cognitive deficits in Alzheimer’s disease. EMBO J..

[81] Ohno Y, Suto S, Yamanaka M, Mizutani Y, Mitsutake S, Igarashi Y (2010). ELOVL1 production of C24 acyl-CoAs is linked to C24 sphingolipid synthesis. Proc Natl Acad Sci USA.

[82] Demirkan A, van Duijn CM, Ugocsai P, Isaacs A, Pramstaller PP, Liebisch G (2012). Genome-wide association study identifies novel loci associated with circulating phospho- and sphingolipid concentrations. PLoS Genet..

[83] Jones L, Holmans PA, Hamshere ML, Harold D, Moskvina V, Ivanov D (2010). Genetic evidence implicates the immune system and cholesterol metabolism in the aetiology of Alzheimer’s disease. PLoS One.

[84] Katsel P, Li C, Haroutunian V (2007). Gene Expression alterations in the sphingolipid metabolism pathways during progression of dementia and Alzheimer’s Disease: a shift toward ceramide accumulation at the earliest recognizable stages of Alzheimer’s disease?. Neurochem Res..

[85] Akram A, Schmeidler J, Katsel P, Hof PR, Haroutunian V (2010). Increased expression of RXR a in dementia: an early harbinger for the cholesterol dyshomeostasis. Mol Neurodegener..

[86] Akram A, Schmeidler J, Katsel P, Hof PR, Haroutunian V (2012). Association of ApoE and LRP mRNA levels with dementia and AD neuropathology. Neurobiol Aging.

[87] Cutler RG, Kelly J, Storie K, Pedersen WA, Tammara A, Hatanpaa K (2004). Involvement of oxidative stress-induced abnormalities in ceramide and cholesterol metabolism in brain aging and Alzheimer’s disease. Proc Natl Acad Sci USA.

[88] He X, Huang Y, Li B, Gong C-X, Schuchman EH (2010). Deregulation of sphingolipid metabolism in Alzheimer’s disease. Neurobiol Aging..

[89] Mielke MM, Haughey NJ, Bandaru VVR, Zetterberg H, Blennow K, Andreasson U (2014). Cerebrospinal fluid sphingolipids, β-amyloid, and tau in adults at risk for Alzheimer’s disease. Neurobiol Aging..

[90] Foley P (2010). Lipids in Alzheimer’s disease: a century-old story. Biochim Biophys Acta..

[91] Di Paolo G, Kim T-W (2011). Linking lipids to Alzheimer’s disease: cholesterol and beyond. Nat Rev Neurosci..

[92] Cedar H, Bergman Y (2009). Linking DNA methylation and histone modification: patterns and paradigms. Nat Rev Genet..

[93] Bernstein BE, Mikkelsen TS, Xie X, Kamal M, Huebert DJ, Cuff J (2006). A bivalent chromatin structure marks key developmental genes in embryonic stem cells. Cell..

[94] Watson CT, Disanto G, Sandve GK, Breden F, Giovannoni G, Ramagopalan SV (2012). Age-associated hyper-methylated regions in the human brain overlap with bivalent chromatin domains. PLoS One..

[95] Meissner A, Mikkelsen TS, Gu H, Wernig M, Hanna J, Sivachenko A (2008). Genome-scale DNA methylation maps of pluripotent and differentiated cells. Nature..

[96] Ohm JE, Baylin SB (2014). Stem cell chromatin patterns: an instructive mechanism for DNA hypermethylation?. Cell Cycle..

[97] Hu S, Wan J, Su Y, Song Q, Zeng Y, Nguyen HN (2013). DNA methylation presents distinct binding sites for human transcription factors. Elife..

[98] Hudry E, Wu HY, Arbel-Ornath M, Hashimoto T, Matsouaka R, Fan Z (2012). Inhibition of the NFAT pathway alleviates amyloid β neurotoxicity in a mouse model of Alzheimer's disease. J Neurosci..

[99] Abdul HM, Sama MA, Furman JL, Mathis DM, Beckett TL, Weidner AM (2012). Cognitive decline in Alzheimer’s disease is associated with selective changes in calcineurin/NFAT signaling. J Neurosci..

[100] Ramanan VK, Kim S, Holohan K, Shen L, Nho K, Risacher SL (2012). Genome-wide pathway analysis of memory impairment in the Alzheimer’s Disease Neuroimaging Initiative (ADNI) cohort implicates gene candidates, canonical pathways, and networks. Brain Imaging Behav..

[101] Bullido MJ, Martínez-García A, Artiga MJ, Aldudo J, Sastre I, Gil P (2007). A TAP2 genotype associated with Alzheimer’s disease in APOE4 carriers. Neurobiol Aging..

[102] Cong W, Cai H, Wang R, Daimon CM, Maudsley S, Raber K (2012). Altered hypothalamic protein expression in a rat model of Huntington’s disease. PLoS One..

[103] Yu J-T, Tan L (2012). The Role of clusterin in Alzheimer’s disease: pathways, pathogenesis, and therapy. Mol Neurobiol..

[104] Szymanski M, Wang R, Bassett SS, Avramopoulos D (2011). Alzheimer’s risk variants in the clusterin gene are associated with alternative splicing. Transl Psychiatry..

[105] Schürmann B, Wiese B, Bickel H, Weyerer S, Riedel-Heller SG, Pentzek M (2011). Association of the Alzheimer’s disease clusterin risk allele with plasma clusterin concentration. J Alzheimers Dis..

[106] Miller JA, Woltjer RL, Goodenbour JM, Horvath S, Geschwind DH (2013). Genes and pathways underlying regional and cell type changes in Alzheimer’s disease. Genome Med..

