# Supplementary figures and images for: Genome-wide12 DNA methylation profiling in the superior temporal gyrus reveals epigenetic signatures associated with Alzheimer’s disease
Source: Genome Med. 2016 Jan 19;8:5. doi: 10.1186/s13073-015-0258-8 (PMC4719699; doi:10.1186/s13073-015-0258-8)

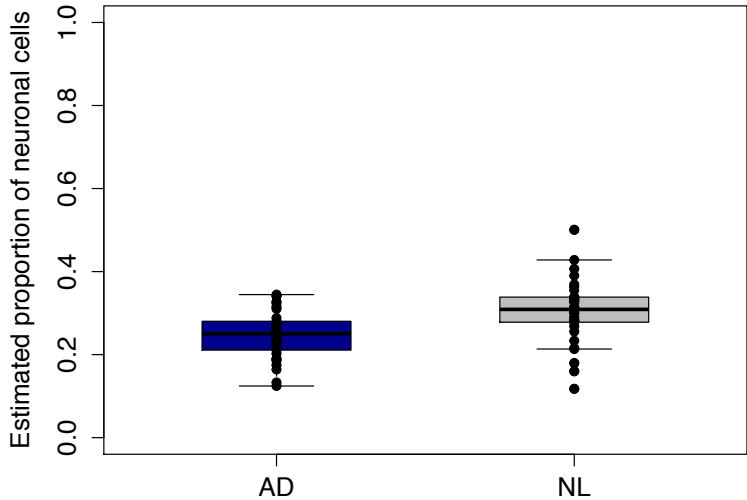

Supplement: Additional file 3: Figure S1. — Estimated neuronal proportions in AD samples are on average lower than those observed in controls. Box plots of per sample neuronal proportions within AD and control sample groups (AD mean = 0.247; control mean = 0.303). Neuronal and glial cell proportions in STG bulk tissue samples were estimated from 450 K methylation profiles of each sample using the CETS R package [54]. A Student’s t-test revealed the difference in neuronal proportions between groups to be significantly different (P = 0.00099), motivating our use of per sample neuronal proportions as a covariate in our regression models for identifying regions of differential methylation. (PDF 65 kb) [file 13073_2015_258_MOESM3_ESM.pdf]

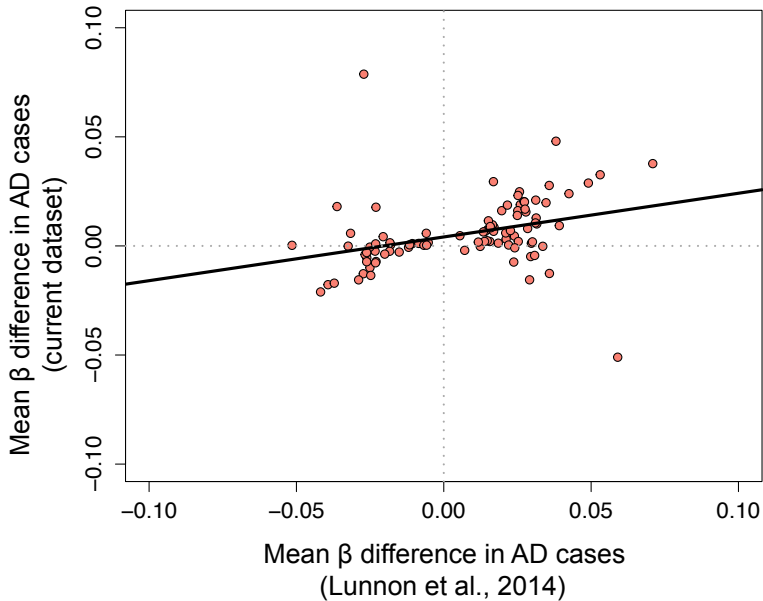

Supplement: Additional file 4: Figure S2. — Comparison of AD-associated methylation changes between two datasets generated from the STG. We compared the estimated mean β-value changes associated with AD status in our dataset (using multiple regression; see “Methods”) to those reported previously for the top 100 differentially methylated CpGs characterized in the STG of an AD discovery cohort by Lunnon et al. [42]. Regression analysis reveals a statistically significant relationship between case–control β-value differences observed in the two studies (r = 0.34; P = 0.00067), with the majority of compared CpGs showing concordant directional changes in methylation associated with AD case status. The red line represents the line-of-best-fit estimated using linear regression. (PDF 98 kb) [file 13073_2015_258_MOESM4_ESM.pdf]

**A**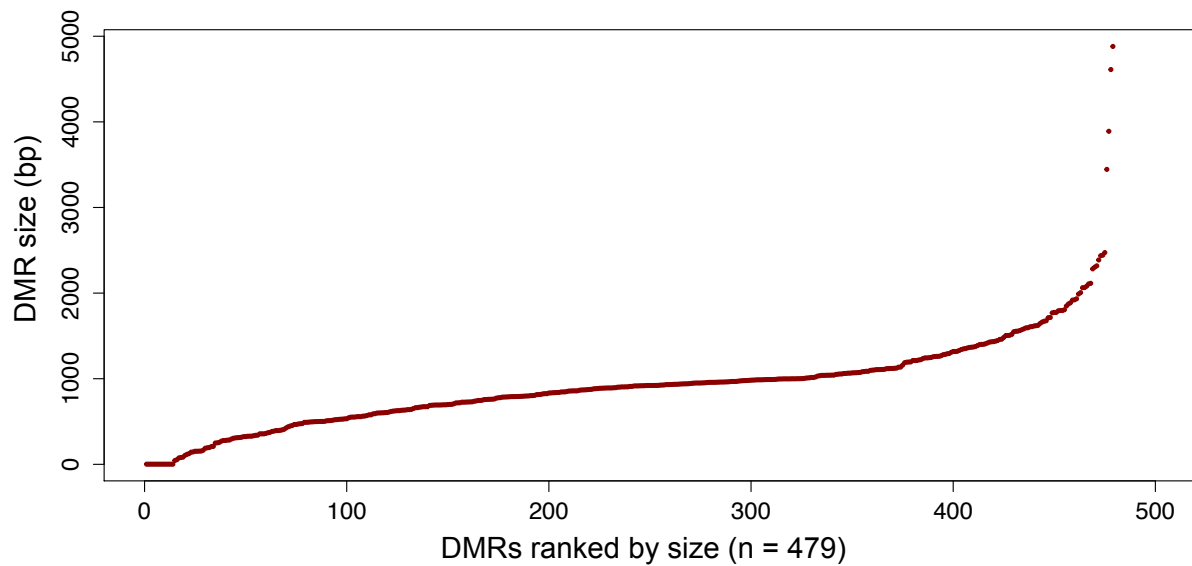**B**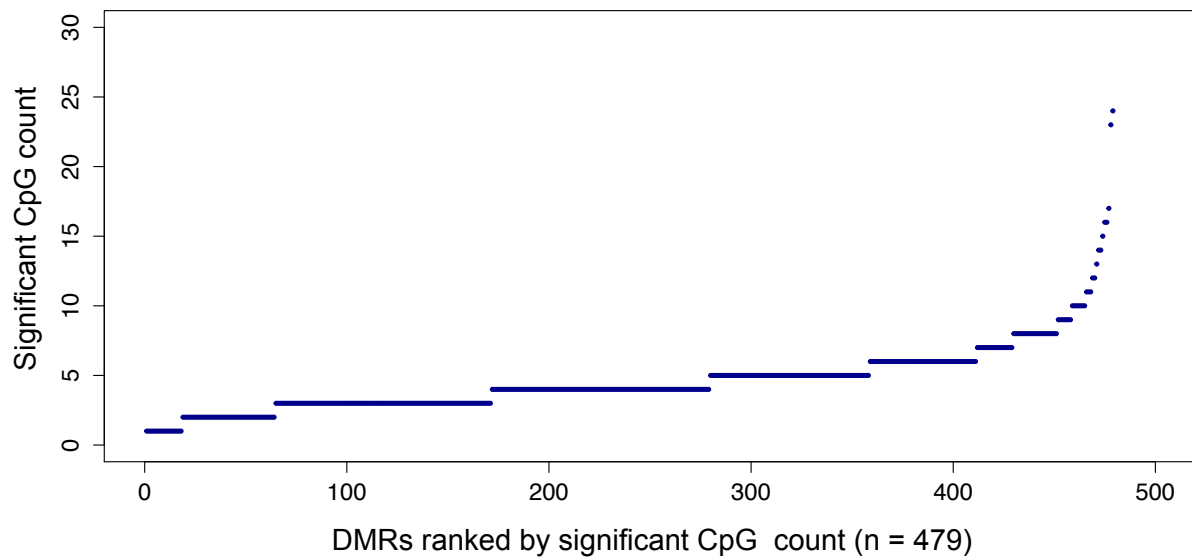

Supplement: Additional file 5: Figure S3. — Summary characteristics of significant DMRs identified in the SGT of AD patients. (A) The length in bp of 479 significant DMRs, ranked by size (minimum = 2 bp; mean = 927 bp; maximum = 4,881 bp). (B) The number of CpGs per DMR that were independently significant by linear regression (P < 0.05; minimum = 1; mean = 4.63; maximum = 24), plotted in ranked order. (PDF 169 kb) [file 13073_2015_258_MOESM5_ESM.pdf]

A

Hyper-DMRs

Hypo-DMRs

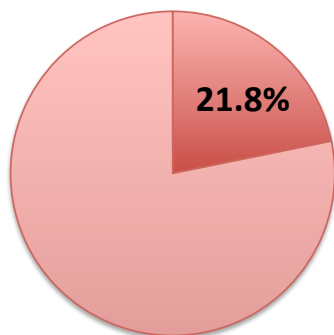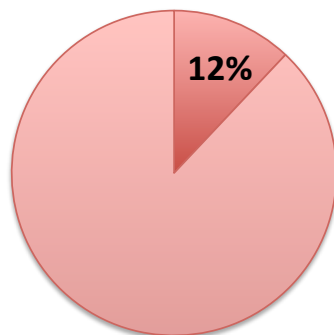

■ significant    ■ non-significant

B

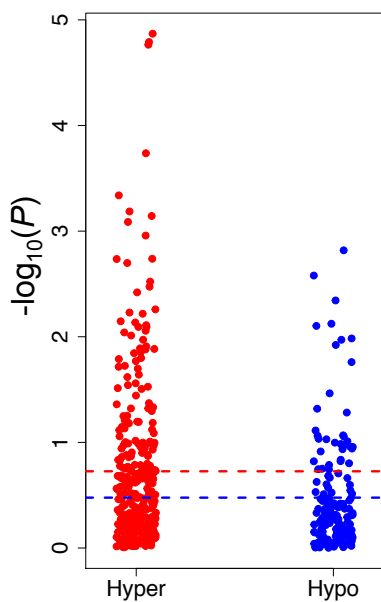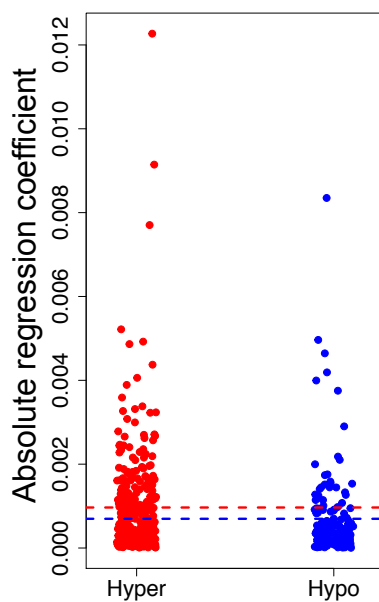

--- Hyper mean    --- Hypo mean

Supplement: Additional file 7: Figure S4. — Significant CpGs within hypermethylated DMRs are enriched for sites associated with aging in controls. (A) The proportion of hypermethylated CpGs that are significantly associated with control sample age is greater among CpGs within AD-associated hypermethylated DMRs. Proportions of significant (P < 0.05, one-tailed) and non-significant CpGs (P > 0.05, one-tailed), as determined by linear regression, within each group (hyper vs. hypo) are indicated. (B) Distributions of –log10 P-values and absolute regression coefficients for effects of sample AOD on CpG methylation, determined using linear regression, after partitioning by DMR status. Hypermethylated CpGs are shown in red (n = 321), and hypomethylated CpGs are shown in blue (n = 158). Means for each metric are indicated by red (hyper) and blue (hypo) dotted lines. (PDF 85 kb) [file 13073_2015_258_MOESM7_ESM.pdf]
