# Supplementary material for: Genome-wide12 DNA methylation profiling in the superior temporal gyrus reveals epigenetic signatures associated with Alzheimer’s disease
Source: Genome Med. 2016 Jan 19;8:5. doi: 10.1186/s13073-015-0258-8 (PMC4719699; doi:10.1186/s13073-015-0258-8)
Supplement: Additional file 12: Figure S5. — Genomic regions of three AD-DMRs encompassing SNPs in LD with AD-GWAS risk variants and brain eQTLs. Panels (A-C) show genomic regions surrounding the DMRs presented in Additional file 11: Table S7, including positions of DMRs, AD-GWAS risk SNPs, brain eQTLs, and RefSeq gene annotations. Red boxes indicate genes whose expression is influenced by identified eQTLs; names of GWAS variant and eQTL SNP IDs are provided below relevant annotations. (DOCX 15 kb) [file 13073_2015_258_MOESM12_ESM.pdf]

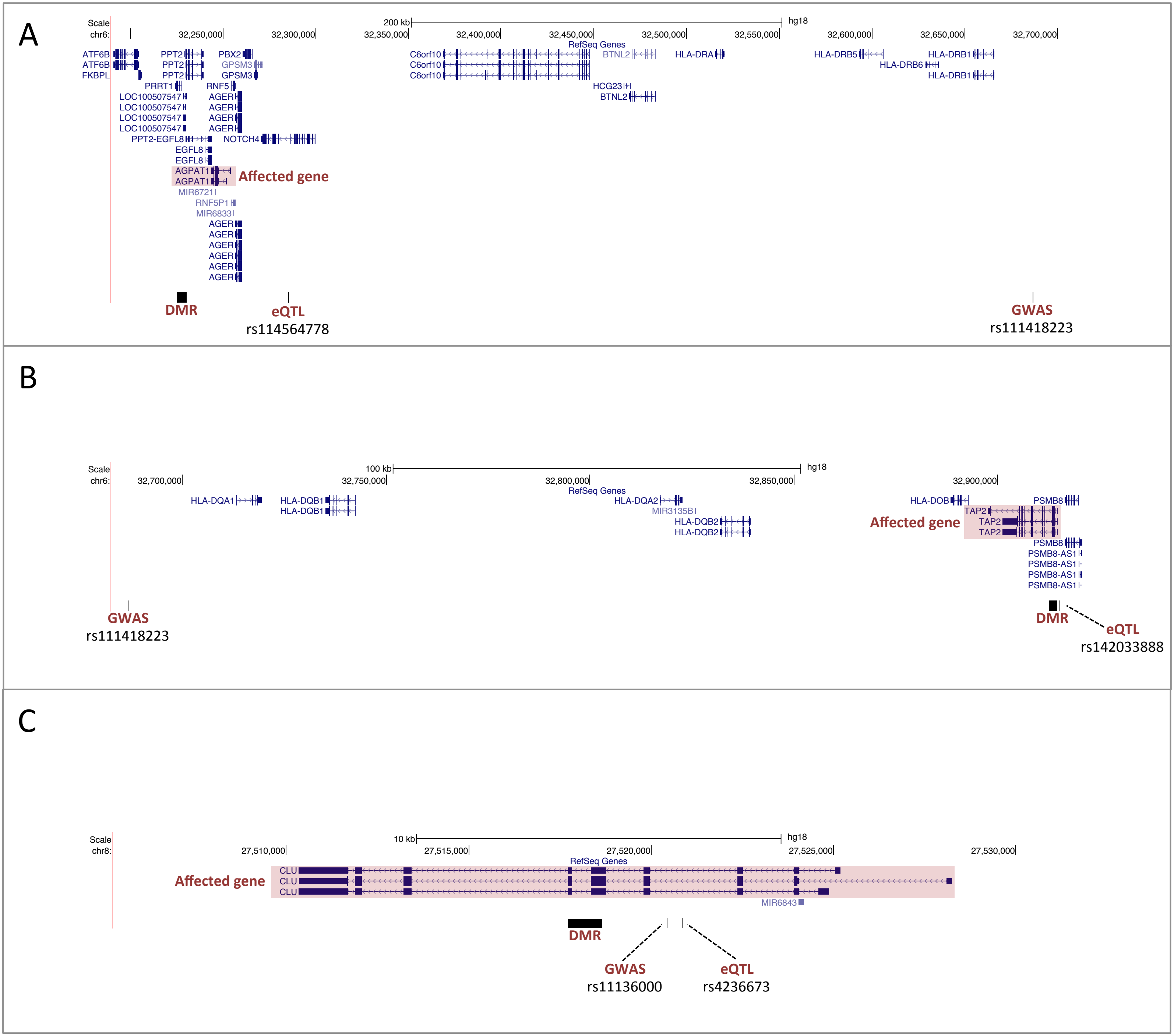
A
Scale
chr6:
200 kb
hg18
32,250,000
32,300,000
32,350,000
32,400,000
32,450,000
32,500,000
32,550,000
32,600,000
32,650,000
32,700,000
RefSeq Genes
ATF6B
FKBPL
PPT2
PBX2
GPSM3
PRRT1
RNF5
LOC100507547
AGER
PPT2-EGFL8
NOTCH4
EGFL8
AGPAT1
Affected gene
MIR6721
RNF5P1
MIR6833
C6orf10
BTNL2
HLA-DRA
HCG23
HLA-DRB5
HLA-DRB6
HLA-DRB1
DMR
eQTL
rs114564778
GWAS
rs111418223
B
Scale
chr6:
100 kb
hg18
32,700,000
32,750,000
32,800,000
32,850,000
32,900,000
RefSeq Genes
HLA-DQA1
HLA-DQB1
HLA-DQA2
MIR3135B
HLA-DQB2
HLA-DOB
PSMB8
TAP2
Affected gene
PSMB8-AS1
GWAS
rs111418223
DMR
eQTL
rs142033888
C
Scale
chr8:
10 kb
hg18
27,510,000
27,515,000
27,520,000
27,525,000
27,530,000
RefSeq Genes
Affected gene
CLU
MIR6843
DMR
GWAS
rs11136000
eQTL
rs4236673
